# Hemiarthroplasty Versus Total Hip Arthroplasty for Recurrent Intertrochanteric Fracture: A Case Report

**DOI:** 10.7759/cureus.88815

**Published:** 2025-07-26

**Authors:** Paul Gerges, Ricardo Rios, Vincent Lee, Mary George, Ralph Rizk

**Affiliations:** 1 Medicine, Edward Via College of Osteopathic Medicine - Carolinas Campus, Spartanburg, USA; 2 Medicine, Edward Via College of Osteopathic Medicine - Auburn Campus, Auburn, USA; 3 Medicine, University of California, Los Angeles, Los Angeles, USA; 4 Orthopedic Surgery, Dr. Ralph Rizk Orthopedics, Jacksonville, USA

**Keywords:** hemiarthroplasty of hip, hip surgery, intertrochanteric fracture, outcomes of hip fracture, total hip arthroplasty

## Abstract

Intertrochanteric fractures in elderly patients present a significant challenge, particularly when prior fixation has failed. Surgical management options include hemiarthroplasty and total hip arthroplasty (THA), with the choice influenced by factors such as patient age, functional status, and bone integrity. Hemiarthroplasty replaces only the femoral head, whereas THA replaces both the femoral head and the acetabulum. As a less invasive option, hemiarthroplasty may be preferred for older, medically frail patients. This case report discusses an 89-year-old man with a history of open reduction and internal fixation (ORIF) for an intertrochanteric fracture who presented with a recurrent fracture and implant failure. Given his advanced age and functional needs, hemiarthroplasty was selected over THA. The procedure was performed using a posterior approach, balancing the benefits of surgical exposure with the risks of dislocation. This case highlights the decision-making process involved in choosing hemiarthroplasty over THA and compares the benefits and limitations of each procedure.

## Introduction

Intertrochanteric fractures account for nearly half of all hip fractures in the elderly and are associated with high morbidity, mortality, and healthcare utilization [1]. These injuries typically result from low-energy trauma, such as a fall from standing height, and are often seen in the setting of osteoporotic bone. Initial treatment usually involves open reduction and internal fixation (ORIF) with a dynamic hip screw or intramedullary nail. However, fixation failure due to nonunion, hardware failure, or repeat trauma is not uncommon in patients with poor bone quality or advanced age [2]. When ORIF fails, conversion to arthroplasty may be necessary to restore mobility and relieve pain. The two main surgical options are hemiarthroplasty and total hip arthroplasty (THA). Hemiarthroplasty replaces the femoral head only, while THA replaces both the femoral and acetabular components. The decision between the two depends on factors such as patient age, activity level, comorbidities, and the condition of the acetabulum.

Hemiarthroplasty is often preferred in medically frail, low-demand patients due to its shorter operative time, lower blood loss, and lower risk of dislocation. THA, while offering superior long-term function and lower risk of acetabular erosion, carries a higher risk of complications and requires longer operative time and recovery [3]. Although several studies compare THA and hemiarthroplasty for femoral neck fractures, there is limited literature on managing failed intertrochanteric fracture fixation with arthroplasty. This case report discusses the surgical management of an 89-year-old man with recurrent intertrochanteric fracture and implant failure, and the rationale for choosing hemiarthroplasty over THA to optimize recovery and reduce surgical risk.

## Case presentation

**Figure 1:**
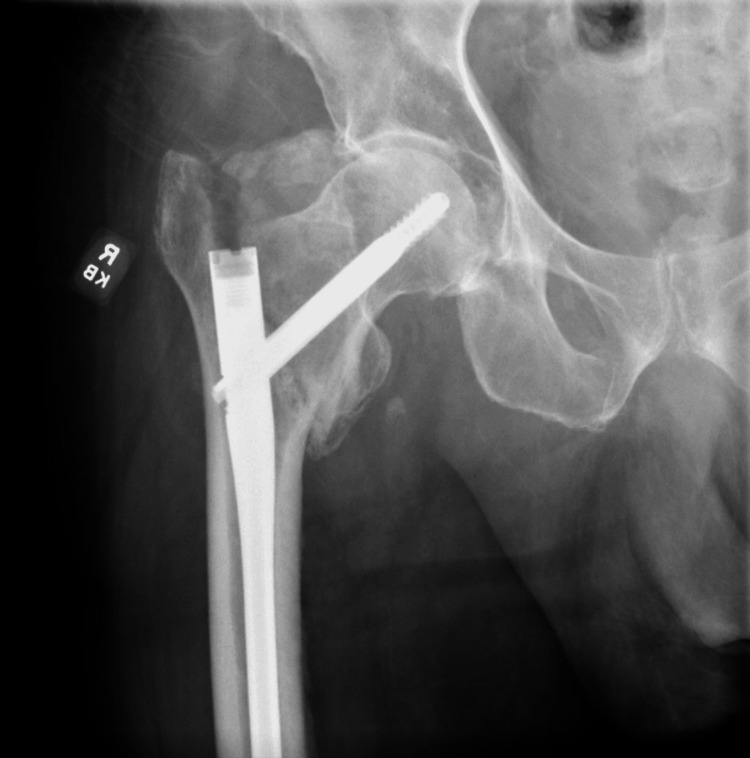
Preoperative anteroposterior radiograph of the right hip Anteroposterior view of the right hip showing a recurrent intertrochanteric fracture with inferior migration of the prior lag screw and subsidence of the intramedullary nail, consistent with implant failure.

An 89-year-old man with a past surgical history of ORIF for an intertrochanteric fracture, repaired with a telescoping lag screw two years prior, presented to the emergency department after a fall. Before the fall, the fracture was noted to be completely healed at a postoperative visit one year prior. After the fall, he reported severe right hip pain and an inability to bear weight. Physical examination revealed external rotation and shortening of the right lower limb, consistent with a hip fracture. Preoperative radiographic evaluation, as shown in Figure 1, demonstrated a recurrent intertrochanteric fracture with downward displacement of the previously implanted nail, confirming implant failure.

Given the patient's advanced age, limited mobility, and lack of acetabular involvement, revision ORIF was not a viable option. Instead, the surgical team considered hemiarthroplasty versus THA to restore function and alleviate pain. After a thorough evaluation, the decision was made to proceed with hemiarthroplasty using a posterior approach. The posterior approach provided adequate exposure for revision surgery while balancing the risk of dislocation with appropriate soft tissue management.

The decision to perform hemiarthroplasty rather than THA was based on several key factors. First, the patient's advanced age and reduced functional demands made hemiarthroplasty a more suitable option, as it provides effective pain relief and allows for early weight-bearing with lower risks of dislocation and surgical complications [3]. Additionally, intertrochanteric fractures primarily affect the proximal femur without compromising the acetabulum, making total joint replacement unnecessary in most cases. Hemiarthroplasty also involves a shorter operative time and reduced intraoperative blood loss compared to THA, which is particularly important in elderly patients with multiple comorbidities [3]. Given these considerations, hemiarthroplasty was chosen as the optimal intervention for this patient.

**Figure 2:**
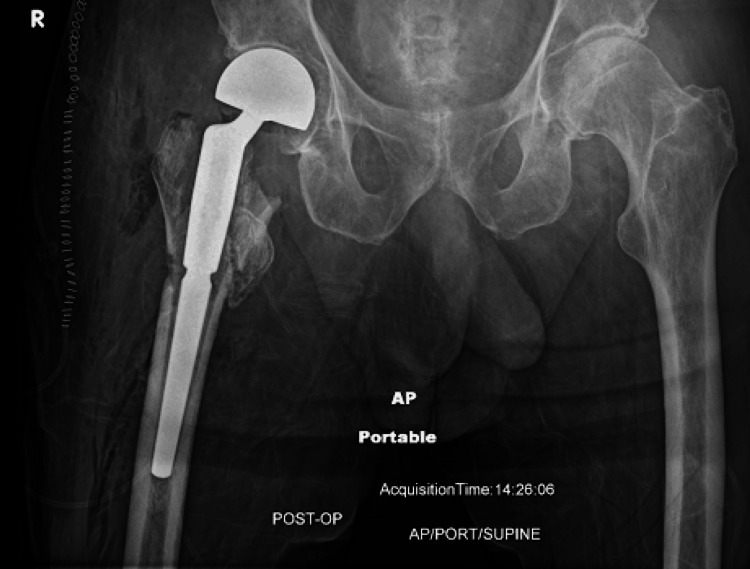
Postoperative anteroposterior radiograph of the right hip Anteroposterior radiograph of the right hip following hemiarthroplasty demonstrating appropriate positioning of the femoral prosthesis without complication.

The procedure was performed without complications, and the patient was started on an early rehabilitation program emphasizing mobilization and fall prevention strategies. Postoperative radiographic evaluation (Figure 2) confirmed appropriate implant positioning without evidence of complications.

## Discussion

Hemiarthroplasty and THA each have distinct advantages and drawbacks. Hemiarthroplasty is associated with lower operative time, decreased blood loss, and a lower risk of postoperative dislocation [4]. It is particularly beneficial in elderly, low-demand patients where acetabular integrity remains intact. However, it has been linked to acetabular wear over time, which can result in pain and reduced function in some patients [5].

Conversely, THA offers superior long-term functional outcomes and reduces the risk of acetabular erosion. It is preferred in active patients or cases where there is pre-existing acetabular pathology [6]. However, THA has a longer operative time and results in more blood loss [7]. Given these considerations, THA is generally reserved for younger, more active patients or those with acetabular involvement.

Complications such as acetabular erosion, femoral stem migration, or component loosening, common to both hemiarthroplasty and total hip arthroplasty, may necessitate conversion or revision to THA [8]. A meta-analysis highlighted that total hip replacement is associated with superior overall results in managing elderly patients, specifically ones with displaced femoral neck fractures [9]. Moreover, patients who initially receive hemiarthroplasty may later require conversion to THA due to chronic pain and reduced mobility.

The functional benefits of THA over hemiarthroplasty are particularly pronounced in active elderly patients. Research indicates that while hemiarthroplasty may allow for earlier weight-bearing and rehabilitation, it often leads to higher rates of certain long-term complications such as acetabular erosion, which can necessitate conversion to THA [10]. In a cohort study, younger patients exhibited a significantly higher conversion rate from hemiarthroplasty to THA, suggesting that age and activity level are critical factors influencing surgical outcomes [11].

Conversely, hemiarthroplasty is often favored in certain clinical scenarios due to its lower initial complication rates and shorter surgical time. It has been noted that the dislocation risk is higher in THA compared to hemiarthroplasty, particularly in the context of revision surgeries [12]. However, the long-term functional outcomes of THA tend to surpass those of hemiarthroplasty, especially in patients who are more physically active or have higher functional demands [13].

## Conclusions

This case highlights the importance of individualized surgical decision-making in elderly patients with recurrent intertrochanteric fractures. Hemiarthroplasty was selected over THA based on the patient's age, functional status, and fracture characteristics. While patient-specific factors remain central, this decision must also be guided by evidence-based practices that compare outcomes, complication rates, and long-term function between procedures. Understanding both clinical guidelines and individualized considerations is essential to optimizing outcomes in geriatric hip fracture management.

## References

[1] Liu F, Chang WJ, Wang X, Gong R, Yuan DT, Zhang YK, Xie WP (2022). Risk factors for prolonged preoperative waiting time of intertrochanteric fracture patients undergoing operative treatment. BMC Musculoskelet Disord.

[2] Liu P, Jin D, Zhang C, Gao Y (2020). Revision surgery due to failed internal fixation of intertrochanteric femoral fracture: current state-of-the-art. BMC Musculoskelet Disord.

[3] Li X, Luo J (2021). Hemiarthroplasty compared to total hip arthroplasty for the treatment of femoral neck fractures: a systematic review and meta-analysis. J Orthop Surg Res.

[4] Macaulay W, Nellans KW, Garvin KL, Iorio R, Healy WL, Rosenwasser MP (2008). Prospective randomized clinical trial comparing hemiarthroplasty to total hip arthroplasty in the treatment of displaced femoral neck fractures: winner of the Dorr Award. J Arthroplasty.

[5] Mahmoud AN, Suk M, Horwitz DS (2024). Symptomatic Acetabular Erosion After Hip Hemiarthroplasty: Is It a Major Concern? A Retrospective Analysis of 2477 Hemiarthroplasty Cases. J Clin Med.

[6] Fan L, Dang X, Wang K (2012). Comparison between bipolar hemiarthroplasty and total hip arthroplasty for unstable intertrochanteric fractures in elderly osteoporotic patients. PLoS One.

[7] Luo S, Qin W, Yu L, Luo R, Liang W (2023). Total hip arthroplasty versus hemiarthroplasty in the treatment of active elderly patients over 75 years with displaced femoral neck fractures: a retrospective study. BMC Musculoskelet Disord.

[8] Poursalehian M, Hassanzadeh A, Lotfi M, Mortazavi SM (2024). Conversion of a Failed Hip Hemiarthroplasty to Total Hip Arthroplasty: A Systematic Review and Meta-Analysis. Arthroplast Today.

[9] Lewis DP, Wæver D, Thorninger R, Donnelly WJ (2019). Hemiarthroplasty vs Total Hip Arthroplasty for the Management of Displaced Neck of Femur Fractures: A Systematic Review and Meta-Analysis. J Arthroplasty.

[10] Liu Y, Chen X, Zhang P, Jiang B (2020). Comparing total hip arthroplasty and hemiarthroplasty for the treatment of displaced femoral neck fracture in the active elderly over 75 years old: a systematic review and meta-analysis of randomized control trials. J Orthop Surg Res.

[11] Moon NH, Shin WC, Do MU, Kang SW, Lee SM, Suh KT (2021). High conversion rate to total hip arthroplasty after hemiarthroplasty in young patients with a minimum 10 years follow-up. BMC Musculoskelet Disord.

[12] Poignard A, Bouhou M, Pidet O, Flouzat-Lachaniette CH, Hernigou P (2011). High dislocation cumulative risk in THA versus hemiarthroplasty for fractures. Clin Orthop Relat Res.

[13] Tang X, Wang D, Liu Y, Chen J, Zhou Z, Li P, Ning N (2020). The comparison between total hip arthroplasty and hemiarthroplasty in patients with femoral neck fractures: a systematic review and meta-analysis based on 25 randomized controlled trials. J Orthop Surg Res.

